# Determination of Factors Associated with Upstage in Atypical Ductal Hyperplasia to Identify Low-Risk Patients Where Active Surveillance May be an Alternative

**DOI:** 10.1245/s10434-024-15041-1

**Published:** 2024-02-22

**Authors:** Alexandra J. E. Greene, Joshua Davis, Jessica Moon, Iram Dubin, Anastasia Cruz, Megha Gupta, Ashkan Moazzez, Junko Ozao-Choy, Esha Gupta, Tejas Manchandia, Babak N. Kalantari, Guita Rahbar, Christine Dauphine

**Affiliations:** 1 Department of Surgery, Harbor-UCLA Medical Center, Torrance, CA USA; 2 Department of Radiology, Harbor-UCLA Medical Center, Torrance, CA USA; 3 Department of Breast Radiology, New York-Presbyterian Hospital/Weill Cornell Medical Center, New York, NY USA; 4 Department of Radiology, Olive View Medical Center, Sylmar, CA USA; 5 Department of Radiology, UCLA Medical Center, Los Angeles, CA USA; 6 Department of Radiology, USC+LAC Medical Center, Los Angeles, CA USA; 7 Department of Radiology, Sharp Coronado Hospital and Healthcare Center, Coronado, CA USA; 8 Department of Radiology, High Desert Regional Health Center, Lancaster, CA USA

**Keywords:** Atypical ductal hyperplasia (ADH), Low-risk cohort, Predictors of upstage

## Abstract

**Background:**

Excision is routinely recommended for atypical ductal hyperplasia (ADH) found on core biopsy given cancer upstage rates of near 20%. Identifying a cohort at low-risk for upstage may avoid low-value surgery. Objectives were to elucidate factors predictive of upstage in ADH, specifically near-complete core sampling, to potentially define a group at low upstage risk.

**Patients and Methods:**

This retrospective, cross-sectional, multi-institutional study from 2015 to 2019 of 221 ADH lesions in 216 patients who underwent excision or active observation (≥ 12 months imaging surveillance, mean follow-up 32.6 months) evaluated clinical, radiologic, pathologic, and procedural factors for association with upstage. Radiologists prospectively examined imaging for lesional size and sampling proportion.

**Results:**

Upstage occurred in 37 (16.7%) lesions, 25 (67.6%) to ductal carcinoma in situ (DCIS) and 12 (32.4%) to invasive cancer. Factors independently predictive of upstage were lesion size ≥ 10 mm (OR 5.47, 95% CI 2.03–14.77, *p* < 0.001), pathologic suspicion for DCIS (OR 12.29, 95% CI 3.24–46.56, *p* < 0.001), and calcification distribution pattern (OR 8.08, 95% CI 2.04–32.00, *p* = 0.003, “regional”; OR 19.28, 95% CI 3.47–106.97, *p* < 0.001, “linear”). Near-complete sampling was not correlated with upstage (*p* = 0.64). All three significant predictors were absent in 65 (29.4%) cases, with a 1.5% upstage rate.

**Conclusions:**

The upstage rate among 221 ADH lesions was 16.7%, highest in lesions ≥ 10 mm, with pathologic suspicion of DCIS, and linear/regional calcifications on mammography. Conversely, 30% of the cohort exhibited all low-risk factors, with an upstage rate < 2%, suggesting that active surveillance may be permissible in lieu of surgery.

Surgical excision is routinely recommended for atypical ductal hyperplasia (ADH) diagnosed on breast core needle biopsy since upstage to malignancy occurs in greater than 20% of cases after excisional biopsy.^1,2^ Further stratification of clinical, radiographic, and pathologic features of ADH at the time of core needle biopsy to determine a low-risk cohort would be ideal to avoid possible overtreatment. However, prior efforts to determine predictors of upstage in patients with ADH are largely single institutional and have been limited by low power given that the incidence of ADH is roughly 3% of all breast biopsies.^3,4^

The selection of factors to study has also been largely focused on data that are readily available upon retrospective chart review, such as patient demographics, core needle gauge, number of biopsy cores taken, and suspicion for ductal carcinoma in situ (DCIS) on the pathology report. The proportion of mammographic lesions sampled, as well as the calcification lesion size, morphology, and/or distribution, have not been consistently recorded in imaging reports, often requiring individual radiographic review to evaluate these factors as predictors of upstage in ADH lesions.

To address the above limitations, the primary aim of this study was to identify clinical, radiologic, pathologic, and procedural factors predictive of cancer upstage in patients diagnosed and treated with ADH within a large multicenter hospital system to delineate a cohort at low upstage risk that may forego surgery. A secondary aim was to categorize individual ADH lesions by the proportion of lesion (mass/calcification) removed during the core needle biopsy procedure to analyze this factor as a predictor of cancer upstage.

## Patients and methods

The protocol for this study was approved by the Los Angeles County Department of Public Health Institutional Review Board (IRB). Strengthening the Reporting of Observational studies in Epidemiology (STROBE) guidelines were utilized in the reporting of this manuscript.^5^

### Study Population

All patients with atypical ductal hyperplasia diagnosed on core needle biopsy from 1 January 2015 to 31 August 2019 within the Los Angeles County Department of Health Services (LADHS) healthcare network were identified. LADHS is compoed of four medical centers (Harbor-UCLA, LAC+USC, Martin Luther King, and Olive View) and serves as a hospital safety net for a predominately urban-underserved, minority population.

Subjects were initially identified by performing an electronic search of breast pathology reports using the phrase “atypi” for the study time interval. Subsequently, patients with atypical lobular hyperplasia or flat epithelial atypia without the presence of ADH, and those with a concurrent diagnosis of breast cancer, were excluded. For this analysis, “cytologic atypia” alone was not included. Excisional biopsy is routinely recommended for all patients with ADH, but may not have been performed if patient declined, left the health system, or was deemed an unsafe surgical candidate. If excisional biopsy was not performed, subjects with fewer than 12 months mammographic follow-up were also excluded from analysis.

### Risk Factors Examined

Clinical, radiologic/procedural, and pathologic factors were recorded and analyzed. Clinical factors included patient age, race/ethnicity, and presence of breast cancer risk factors (personal or family history of breast and/or ovarian cancer and Tyrer–Cuzick breast cancer risk assessment score). Race/ethnicity was collected from the electronic medical record (EMR) and represents self-reported data recorded at the time of patient registration. Race/ethnicity was categorized into the following groups: Asian, Black, Hispanic White, Non-Hispanic White, and other. Personal and family (at least one primary or secondary relative) history of breast and/or ovarian cancer was collected from mammogram intake surveys. Tyrer–Cuzick scores are routinely calculated using the breast cancer risk assessment program embedded within the MagView (Fulton, Maryland) mammography tracking and reporting software for all LADHS patients undergoing screening mammography, and these values were abstracted from the prebiopsy mammography report for this analysis (prior to the diagnosis of ADH).

**Fig 1:**
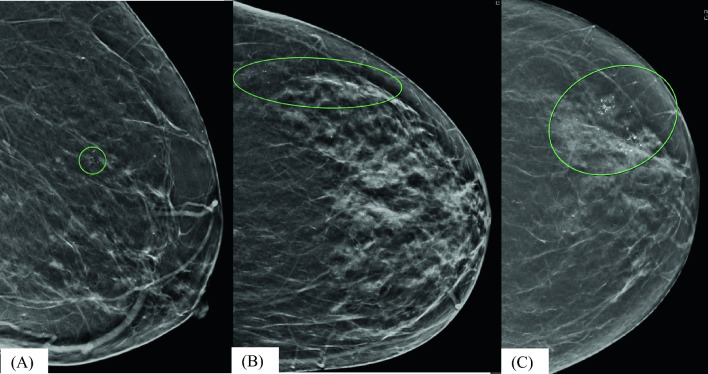
Radiographic examples of calcification distribution; **A** grouped: right mediolateral (ML) view, a 62-year-old woman presented with grouped calcifications in the right breast and stereotactic biopsy yielded ADH and no evidence of malignancy on excisional biopsy; **B** linear: left cranial caudal (CC) view; a 54-year-old woman presented with calcifications with a linear distribution in the left outer breast, stereotactic biopsy yielded ADH, and there was no evidence of malignancy on excisional biopsy; and **C** regional: left CC view, a 65-year-old woman presented with calcifications with regional distribution in the left outer breast, stereotactic biopsy yielded ADH, and she was upgraded to DCIS and invasive ductal carcinoma (IDC) on excisional biopsy

Radiologic and procedural factors collected were lesion size, lesion morphology and distribution on mammography, core biopsy needle caliber (small, 11–18 gauge; large, 7–9 gauge), and type of image guidance (stereotactic versus ultrasound). Existing pre and post-biopsy mammograms for all subjects were prospectively reexamined by a board-certified breast radiologist on high-resolution monitors from stored electronic image files to confirm lesion size (single greatest dimension of calcification/mass), calcification morphology (benign, amorphous, coarse heterogeneous, fine pleomorphic, fine linear, or fine linear branching), calcification distribution (diffuse, regional, grouped, linear, or segmental), and to visually estimate the proportion of lesion biopsied (10% or less of the lesion removed, 11–50% of lesion removed, 51–89% of lesion removed, and 90% or greater of the lesion removed). Each radiologist performing the reexamination of films underwent a brief training on definitions and coding of lesional size and proportion biopsied. Calcification morphology and distribution patterns (Fig. 1) were classified using the descriptors outlined by the American College of Radiology.^6^ Pathologic factors included histologic results from the core needle biopsy report (suspicion of ductal carcinoma in situ, number of ADH foci, and presence of concurrent lobular or flat epithelial atypia).

### Outcomes

The primary outcome measure for this study was the determination of factors predictive of ADH upstage to malignancy (invasive and/or noninvasive) to identify a low-risk cohort. Upstage was determined to have occurred if excisional biopsy demonstrated an invasive or in situ cancer, or in cases where excision was not performed, subsequent mammographic follow up detected a suspicious lesion that ultimately revealed DCIS or invasive cancer on repeat core biopsy. A “low upstage risk” cohort was defined as the absence of all statistically significant independent predictors of upstage. The secondary outcome was determining whether near-complete sampling of an ADH lesion during core biopsy is predictive of cancer upstage.

### Statistical Analysis

Bivariate analysis was used to determine factors that were associated with upstage to cancer. A two-sided *p* < 0.05 was considered statistically significant. Continuous data were compared using the Student’s *t*-test or Levene’s test for equality of variances and categorical data were compared using Pearson *χ*^2^ and Fisher’s exact tests as appropriate. Classification and regression tree (CART) analysis was used to elucidate possible cutoff points for variables, such as size of lesion and patient’s age. CART analysis is a tree-building model that applies a dichotomous split in the dependent or explanatory variable, in this case upstage to cancer, on the basis of independent predictors, in this case the size of the lesion and the age of the patient at biopsy. This technique demonstrates the relationship of variables in terms of explanatory power and variance, and in turn, is useful for informing further intervention. Multivariable regression was then used to validate any variable with *p* < 0.1 in initial bivariate analysis. For the portion of the cohort not exhibiting high-risk factors, the ADH upstage rate was then calculated and further characterization of this group was defined. SPSS version 24 (IBM Corp, Armonk, New York) was used to perform statistical analysis.

## Results

Of the 238 ADH lesions identified, 17 (7.1%) were excluded as they did not undergo excisional biopsy and did not have at least 12 months of mammographic follow-up. The final cohort was composed of 221 lesions in 216 patients, 206 (93.2%) of which had an excisional biopsy and 15 (6.8%) of which had a minimum of 12 months of close mammographic follow-up after core biopsy. The mean age of the entire cohort was 54.5 years (range 28–76 years) and race distribution was 26 (11.8%) Asian, 28 (12.7%) Black or African American, 132 (59.7%) Hispanic or Latinx, 17 (7.7%) Non-Hispanic White, and 18 (8.1%) other. Patient, radiologic, and pathologic characteristics for the excisional biopsy group versus the close mammographic follow-up group are presented in Table 1 and were not found to be significantly different (all *p* < 0.05). Hematoma was visible at the core biopsy site on 53 (24.1%) post-biopsy mammograms, 18 (8.1%) of which were noted to obscure the biopsy site. Clip migration greater than 1 cm away from the biopsy site was reported in 16 (7.2%) cases.

**Table 1:** Radiologic, pathologic, clinical, and procedural factors of cohort subgrouped by intervention and by outcome

Factors of core biopsy	Cohort subgroup by intervention (*n* = 221)		Significance (*p* value)	Cohort subgroup by outcome (*n* = 221)		Significance (*p* value)
	Excisional biopsy (*n* = 206)	Mammographic follow-up (*n* = 15)		Upstage (*n* = 37)	No upstage (*n* = 184)	
**Mean age (years) [SD]**	54 [± 8.9]	56.8 [± 6.3]	*p* = 0.064	54.6 [± 9.2]	54.1 [± 8.7]	*p* = 0.531
**Race**			*p* = 0.712			*p* = 0.825
Asian	25 (12.1%)	1 (6.7%)		6 (16.2%)	20 (10.9%)	
Black or African American	26 (12.6%)	2 (13.3%)		4 (10.8%)	24 (13.0%)	
Hispanic or Latinx	122 (59.2%)	10 (66.7%)		20 (54.1%)	112 (60.9%)	
Non-Hispanic White	15 (7.3%)	2 (13.3%)		3 (8.1%)	14 (7.6%)	
Other	18 (8.7%)	0		4 (10.8%)	14 (7.6%)	
**Mammographic density**			*p* = 0.082			*p* = 0.677
Fatty	3 (1.5%)	1 (6.7%)		0	4 (2.2%)	
Scattered	64 (31.1%)	8 (53.3%)		14 (37.8%)	58 (31.5%)	
Heterogeneous	124 (60.2%)	6 (40.0%)		22 (59.5%)	108 (58.7%)	
Extreme	15 (7.3%)	0		1 (2.7%)	14 (7.6%)	
**Lesion size (median, mean; mm) [SD]**	10, 12.7 [± 11.8]	7, 10.1 [± 9.7]	*p* = 0.171	12, 19.0 [± 15.6]	8, 11.2 [± 10.2]	*p* = 0.003
**Proportion removed at biopsy**			*p* = 0.306			*p* = 0.614
10% or less	70 (34.0%)	2 (13.3%)		14 (37.8%)	58 (31.5%)	
11–50%	50 (24.3%)	6 (40.0%)		11 (29.7%)	45 (24.5%)	
51–89%	47 (22.8%)	4 (26.7%)		6 (16.2%)	45 (24.5%)	
90% or greater	39 (18.9%)	3 (20.0%)		6 (16.2%)	36 (19.6%)	
**Calcification morphology**			*p* = 0.264			*p* = 0.125
Benign	14 (6.8%)	0 (0%)		2 (5.4%)	12 (6.5%)	
Amorphous	37 (18.0%)	5 (33.3%)		4 (10.8%)	38 (20.7%)	
Coarse heterogeneous	45 (21.8%)	4 (26.7%)		7 (18.9%)	42 (22.8%)	
Fine pleomorphic	39 (18.9%)	0 (0%)		11 (29.7%)	28 (15.2%)	
Fine linear (branching)	2 (1.0%)	0 (0%)		1 (2.7%)	1 (0.5%)	
Calcifications not the target	68 (33.0%)	6 (40.0%)		12 (32.4%)	62 (33.7%)	
**Calcification distribution**			*p* = 0.772			*p* = 0.002
Diffuse	3 (1.5%)	0 (0%)		0 (0%)	3 (1.6%)	
Regional	13 (6.3%)	1 (6.7%)		7 (18.9%)	7 (3.8%)	
Grouped	105 (51.0%)	7 (46.7%)		12 (32.4%)	100 (54.3%)	
Linear	11 (5.3%)	0 (0%)		5 (13.5%)	6 (3.3%)	
Segmental	6 (2.9%)	1 (6.7%)		1 (2.7%)	6 (3.3%)	
Calcifications not the target	68 (33.0%)	6 (40.0%)		12 (32.4%)	62 (33.7%)	
**Mass/asymmetry at biopsy site**	68 (33.0%)	7 (46.7%)	*p* = 0.314	13 (35.1%)	62 (33.7%)	*p* = 0.848
**Core biopsy histology**			*p* = 1.00			*p* = 0.236
Pure ADH	184 (89.3%)	14 (93.3%)		31 (83.8%)	167 (90.8%)	
ADH + FEA or ALH	22 (10.7%)	1 (6.7%)		6 (16.2%)	17 (9.2%)	
**DCIS suspected on pathology**	14 (6.8%)	0 (0%)	*p* = 0.606	7 (18.9%)	7 (3.8%)	*p* = 0.003
**Method of biopsy**			*p* = 0.519			*p* = 0.829
Ultrasound	70 (34.0%)	7 (46.7%)		14 (37.8%)	63 (34.2%)	
Stereo	133 (64.6%)	8 (53.3%)		23 (62.2%)	118 (64.1%)	
MRI	3 (1.5%)	0 (0%)		0 (0%)	3 (1.6%)	
**Needle size (gauge)**			*p* = 0.334		*p* = 0.651	
Small (18, 14, 13, 12, 11)	97 (47.1%)	9 (60.0%)		19 (51.4%)	87 (47.3%)	
Large (7, 8, 9)	109 (52.9%)	6 (40.0%)		18 (48.6%)	97 (52.7%)	
**Personal history of cancer**			*p* = 0.106			*p* = 0.330
None	186 (90.3%)	14 (93.3%)		32 (86.5%)	168 (91.3%)	
Breast cancer	19 (9.2%)	0 (0%)		5 (13.5%)	14 (7.6%)	
Ovarian cancer	1 (0.5%)	1 (6.7%)		0 (0%)	2 (1.1%)	
**Family history of cancer**			*p* = 0.772			*p* = 0.046
None	157 (76.2%)	11 (73.3%)		33 (89.2%)	135 (73.4%)	
Breast cancer	48 (23.3%)	4 (26.7%)		4 (10.8%)	48 (26.1%)	
Ovarian cancer	1 (0.5%)	0 (0%)		0 (0%)	1 (0.5%)	
**Tyrer–Cuzick score**			*p* = 0.428			*p* = 0.383
Average risk (< 15)	120 (58.3%)	13 (86.7%)		22 (59.5%)	111 (60.3%)	
Elevated risk (≥ 15)	42 (20.4%)	1 (6.7%)		3 (8.1%)	40 (21.7%)	
Unknown	44 (21.4%)	1 (6.7%)		12 (32.4%)	33 (17.9%)	

*mm* millimeters, *ADH* atypical ductal hyperplasia, *FEA* flat epithelial atypia, *ALH* atypical lobular hyperplasia, *DCIS* ductal carcinoma in situ, *MRI* magnetic resonance imaging

ADH upstage occurred in 37 (16.7%) lesions, 25 (67.6%) DCIS and 12 (32.4%) invasive cancer, and all were in the excisional biopsy group. With an average follow-up interval of 32.6 months (range 12–60 months), patients in the mammographic follow-up group did not have any subsequent imaging that noted abnormalities at the biopsy site (i.e., no upstage). Upstage rates associated with individual factors are listed in Table 2.

**Table 2:** Upstage rates by radiologic, pathologic, and clinical factors and significance by bivariate and multivariate analysis

Factors	Upstage rate	Bivariate analysis	Multivariate analysis
**Mammographic density**		*p* = 0.677	NS
Fatty	0.0%		
Scattered	19.4%		
Heterogeneous	16.9%		
Extreme	6.7%		
**Lesion size (mm)**		*p* = 0.003	OR 1.04, 95% CI 1.01–1.08, *p* = 0.018
≥ 10 mm	26.6%	*p* = 0.007	OR 5.47, 95% CI 2.03–14.77, *p* < 0.001
< 10 mm	7.1%		**
**Proportion removed at biopsy**		*p* = 0.614	NS
< 50%	19.5%	*p* = 0.193	NS
≥ 50%	12.9%		
< 90%	17.3%	*p* = 0.636	NS
≥ 90%	14.3%		
**Calcification morphology**		*p* = 0.125	NS
Benign	14.3%		
Amorphous	9.5%		
Coarse heterogeneous	14.2%		
Fine pleomorphic	28.2%		
Fine linear (branching)	50.0%		
Calcifications not the target	16.0%		
**Calcification distribution**		*p* = 0.002	*p* = 0.008
Diffuse	0.0%		OR 0, *p* = 0.999
Regional	50.0%		OR 8.08, 95% CI 2.04–32.00, *p* = 0.003
Grouped	10.7%		**
Linear	45.5%		OR 19.28, 95% CI 3.47–106.97, *p* < 0.001
Segmental	14.3%		OR 0.87, 95% CI 0.09–8.35 *p* = 0.9
Calcifications not the target	16.2%		OR 1.55, 95% CI 0.59–4.04, *p* = 0.37
**Mass at biopsy site**	17.3%	*p* = 0.848	NS
**Core biopsy histology**		*p* = 0.236	NS
Pure ADH	15.6%%		
ADH + FEA or ALH	26.1%		
**DCIS suspected on pathology**	50.0%	*p* = 0.003	OR 12.29, 95% CI 3.24–46.56, *p* < 0.001
**Method of biopsy**		*p* = 0.829	NS
Ultrasound	18.2%		
Stereo	16.3%		
MRI	0.0%		
**Needle size (gauge)**		*p* = 0.651	NS
Small (18, 14, 13, 12, 11)	17.9%		
Large (7, 8, 9)	15.7%		
**Personal history of cancer**		*p* = 0.330	NS
None	16.0%		
Breast cancer	26.3%		
Ovarian cancer	0.0%		
**Family history of cancer**		*p* = 0.008	OR 0.14, 95% CI 0.03–0.59, *p* = 0.010
None	19.6%		
Breast cancer	7.7%		
Ovarian cancer	0.0%		
**Tyrer–Cuzick score**		*p* = 0.383	NS
Average risk (< 15)	16.5%		
Elevated risk (≥ 15)	7.0%		
Unknown	26.7%		

*mm,* millimeter, *ADH* atypical ductal hyperplasia, *DCIS* ductal carcinoma in situ, *NS* not significant, ** reference value, *OR* odds ratio

On bivariate analysis of the final dataset (*n* = 221), the following factors demonstrated a significant correlation with ADH upstage: lesion size [upstage mean 19.0 mm (SD ± 15.6 mm) versus non-upstage mean 11.2 mm (± 10.2 mm), *p* = 0.003], calcification distribution pattern (upstage rates 50% for “regional,” 45.5% “linear,” 14.3% “segmental,” 10.7% “grouped,” and 0% “diffuse”; *p* = 0.002), and pathologic suspicion of DCIS (upstage rate 50%; *p* = 0.003). Family history of breast cancer was statistically significant with a negative correlation to upstage rate (upstage 7.7% for family history of breast cancer versus 19.6% with negative family history; *p* = 0.046). Proportion of lesion biopsied was not significantly correlated (upstage rate for near-complete sampling ≥ 90% was 14.3% versus 17.3% for < 90% sampled, *p* = 0.636; upstage rate for ≥ 50% sampled was 12.9% versus 19.5% for < 50% sampled, *p* = 0.193). Two factors could not be analyzed owing to the extent of missing data in radiology and pathology reports: number of cores removed during the percutaneous biopsy procedure (missing 89%) and number of ADH foci seen on core pathology (missing 63%). Using CART analysis, size ≥ 10 mm was a significant cutoff point in upstage rates (26.6% upstage in lesions ≥ 10 mm versus 7.1% upstage in lesions < 10 mm, *p* = 0.007), while no cutoff point existed for patient age. A summary of the significance of individual factors on univariate and multivariable analyses are included in Table 2.

Multivariable analysis validated an independent correlation with ADH upstage in three factors: lesion size ≥ 10 mm [odds ratio (OR) 5.47, 95% CI 2.03–14.77, *p* < 0.001], calcification distribution pattern (OR 8.08, 95% CI 2.04–32.00, *p* = 0.003 for “regional”; OR 19.28, 95% CI 3.47–106.97, *p* < 0.001 for “linear”), and DCIS suspected on core biopsy (OR 12.29, 95% CI 3.24–46.56, *p* < 0.001). Family history of cancer was also independently associated with upstage to cancer, although inversely proportional (OR 0.14, 95% CI 0.03–0.59, *p* = 0.008).

When comparing the 25 cases that upstaged to DCIS with the 12 cases that upstaged to invasive cancer, 100% of the invasive upstages were ≥ 10 mm on prebiopsy mammogram (versus 68% of DCIS upstages), “pathologic suspicion of DCIS” was associated with 20% of DCIS upstages (versus 17% of invasive), “grouped” calcification distribution pattern was seen in 36% of DCIS upstage (versus 25% invasive), “regional” or “linear” pattern was noted in 41% of invasive upstage (versus 28% DCIS) and similar rates of < 50% lesion sampled at core biopsy were demonstrated for DCIS and invasive upstages (67% and 68%, respectively).

Of 221 total ADH cases in the study, 65 (29.4%) had ADH lesions smaller than 10 mm, with a non-regional/non-linear calcification distribution pattern, and no suspicion of DCIS on core biopsy, comprising a “low-risk” group. Among these 65 patients, the upstage rate was 1.5% (*n* = 1). The average lesion size among “low-risk” patients was 5.1 mm, and the calcification distribution pattern was predominantly “grouped.”

## Discussion

Published investigations of predictors of upstage in ADH lesions number in the several hundred, with only a few reporting on cohorts larger than 200 subjects, and nearly all being retrospective analyses of data limited to what is available in the patient record.^4,7–10^ In this multicenter study of 221ADH lesions, upstage to cancer was seen in 17%, and factors predictive of upstage were lesion size of 10 mm or larger, pathologic suspicion for presence of DCIS on core specimens, and regional/linear calcification distribution patterns on diagnostic mammography. Near-complete excision of the mammographic lesion by core biopsy trended toward, but did not significantly correlate with, upstage in this series. Almost a third of the patient cohort did not exhibit any of the three high-risk factors, and the upstage rate in this group was only 1.5%, suggesting that a low-risk cohort can be identified among patients diagnosed with ADH on core biopsy.

The association of size of the mammographic lesion (calcifications and/or mass) with cancer upstage is unsurprising given that sampling error increases with the size of the abnormality undergoing biopsy. In the present study, every 1 mm increase was associated with a 4% increase in the odds of upstage. Others have documented a similar association between lesion size and upstage.^11^ However, the size of an area of calcifications on a mammogram is often not documented on imaging reports as is done for mass lesions, and therefore may be understudied in existing literature. In the present study, in which we found 10 mm to be an important cutoff point, radiologist reexamination of digitally stored pre-biopsy images was required to obtain and analyze this factor. Lustig et al.^12^ noted that mammographic and/or ultrasonic size larger than 5 mm was associated with upstage in 290 patients. Further delineation of size cutoffs that discriminate patients at low and high risk for upstage is warranted in future studies.

Data from this study show that calcification characteristics (distribution and possibly morphology) may be important in identifying patients at risk of upstage. A strong positive correlation was demonstrated between the distribution pattern of mammographic calcifications and cancer upstage, with “regional” and “linear” patterns exhibiting high upstage rates of 50% and 45%, respectively, compared with a rate of 11% for “grouped” calcifications. This finding can be explained by the geometric impracticability of obtaining an adequate sample of a linearly or regionally distributed abnormality using a rotational biopsy device that is positioned at static coordinates. Moreover, while not statistically significant in our study, fine pleomorphic and fine branching/linear calcification morphologies had an upstage rate of 28% and 50%, respectively, compared with just 14% for both “coarse” and “benign” morphologies, suggesting that this factor may also be important. Hoang et al.^13^ described a similar finding regarding calcification morphology and distribution pattern and upstage rates. A standardized lexicon of descriptors for the reporting of calcifications seen on mammography has now been outlined by the American College of Radiology, making these factors much more readily available.^6^

Near-complete lesional sampling, defined in our study as removal of at least 90% of the radiographic target during core biopsy, should intuitively result in lower rates of underestimation and therefore low rates of cancer upstage on excision. Examining the effect of near-complete sampling on ADH upstage rates required secondary review of pre and post-biopsy mammograms by a breast imager, as this is not documented routinely on post-procedure imaging reports. While this factor was not statistically significant in our analysis, there was a trend toward lower upstage with near-complete removal, occurring in 14% of lesions compared with a rate of 17% when less than 90% of the visible lesion was removed at biopsy. A pooled subgroup analysis of 14 studies examining complete removal of the mammographic visible ADH lesion at biopsy also noted that the upstage rate remained 14%, albeit lower than the 29% upstage reported overall for ADH lesions.^7^ In contrast, Lustig et al.^12^ noted that “incomplete removal” of calcifications correlated with upstage and included this factor in their risk calculator. Though whether removal of the visible lesion at the time of core biopsy substantially lowers upstage rates appears controversial, it may still be important when trying to identify patients in whom active surveillance is permissible. ADH is characterized as a nonobligate precursor to noninvasive and invasive breast cancer, and as such, removing the mammographic or ultrasonographic lesion during core biopsy may still have clinical benefit in longer-term ADH management if foregoing excision.^14^

In this investigation, 7% of the cohort did not undergo excision and instead were observed for an average of 32.6 months with close mammographic follow-up. No mammographic abnormalities suggestive of upstage to malignancy were detected at the biopsied area in any of the 15 mammographically observed patients. Other studies, including an active observation cohort, report upstage rates of 2–8% with close mammographic follow-up alone.^15–17^ The reason to forego surgical resection was not captured in this dataset, but the predominance of low-risk factors among this group suggests that there may have been some consideration of low upstage risk made at the time of surgical evaluation. Though not statistically significant, the patients in the active surveillance group were slightly older, with less mammographic density, smaller lesions (median 7 mm versus 10 mm in the excision group), coarse heterogenous calcification morphology as opposed to fine pleomorphic, no cases of suspected DCIS, and more often had lower Tyrer–Cuzick scores compared with those who underwent excision of ADH.

Identifying a subgroup of patients with ADH diagnosed on core biopsy who are at low risk for cancer upstage is advantageous to avoid unnecessary and costly surgical procedures and overtreatment of individuals who will derive little benefit from excision. Patients who are poor surgical candidates owing to comorbidities are particularly poised to benefit. Our study and other published calculators and nomograms have identified cohorts with less than 2% upstage risk, which can aid in patient counseling and guide shared decision-making.^10,12,18^ The duration and frequency of close mammographic observation as a means of active surveillance for patients who do not undergo surgical excision is yet to be determined. Active surveillance should likely include at least 24 months of diagnostic mammograms performed every 6 months. Just as those who undergo excision, patients in an active surveillance group should be prescribed chemoprevention for 60 months.^19^

This study has several limitations, including the retrospective design. Tyrer–Cuzick scores and personal or family history data may have been incorrectly and/or incompletely recorded in the EMR as patients may not have been aware of their family history, may have been confused about different anatomical organs, may have had language translational difficulties, or the information may have been misinterpreted by mammographers recording the data. Moreover, interobserver variability in the determination of calcification size, characteristics, and proportion biopsied may have occurred, but each radiologist in the study received training prior to reviewing images to help reduce the occurrence. Furthermore, while radiologists were not specifically told which cases were upstaged when images were reviewed, they were not blinded from becoming aware of the upstage while viewing the imaging files. The strengths of this study include the large multi-institutional cohort, inclusion of non-excision group, and re-review of digital images by breast radiologists to stratify patients according to the proportion of calcifications removed and confirm lesion characteristics for improved accuracy of analysis.

## Conclusions

In a cohort of more than 200 patients with ADH, this multi-institutional study demonstrated an upstage rate to cancer just below 20%. Three factors associated with upstage were identified (size of the prebiopsy mammographic and/or ultrasonographic lesion 10 mm or greater, pathologic suspicion for cancer on core pathology, and a suspicious pattern of calcification distribution) and may help in further refinement of risk calculation tools. Absence of all three high-risk factors was seen in 30% of the cohort, and the upstage rate among this group was under 2%, suggesting that active surveillance as an alternative to surgery may be permissible. Future prospective studies to validate the findings of this study and the safety of close mammographic surveillance in lieu of surgery are warranted.
